# Long-term fungal inoculation of *Ficus sycomorus* and *Tectona grandis* woods with *Aspergillus flavus* and *Penicillium chrysogenum*

**DOI:** 10.1038/s41598-023-37479-1

**Published:** 2023-06-28

**Authors:** Maisa M. A. Mansour, Wafaa A. Mohamed, Ahmed A. A. El-Settawy, Martin Böhm, Mohamed Z. M. Salem, Marwa G. S. Farahat

**Affiliations:** 1 Department of Conservation and Restoration, Faculty of Archaeology, Cairo University, Giza, 12613 Egypt; 2 Forestry and Wood Technology Department, Faculty of Agriculture (El-Shatby), Alexandria University, Alexandria, 21545 Egypt; 3 Department of Materials Engineering and Chemistry, Faculty of Civil Engineering, Czech Technical University in Prague, Thákurova 7, 166 29 Prague 6, Czech Republic

**Keywords:** Biological techniques, Microbiology, Materials science

## Abstract

In the current study, two molds, *Aspergillus flavus* (ACC# LC325160) and *Penicillium chrysogenum* (ACC# LC325162) were inoculated into two types of wood to be examined using scanning electron microscopy-energy dispersive X-ray (SEM–EDX) and computerized tomography (CT) scanning. *Ficus sycomorus*, a non-durable wood, and *Tectona grandis*, a durable wood, were the two wood blocks chosen, and they were inoculated with the two molds and incubated for 36 months at an ambient temperature of 27 ± 2 °C and 70 ± 5% relative humidity (RH). The surface and a 5-mm depth of inoculated wood blocks were histologically evaluated using SEM and CT images. The results showed that *A. flavus* and *P. chrysogenum* grew enormously on and inside of *F. sycomorus* wood blocks, but *T. grandis* wood displayed resistance to mold growth. The atomic percentages of C declined from 61.69% (control) to 59.33% in *F. sycomorus* wood samples inoculated with *A. flavus* while O increased from 37.81 to 39.59%. *P. chrysogenum* caused the C and O atomic percentages in *F. sycomorus* wood to drop to 58.43%, and 26.34%, respectively. C with atomic percentages in Teak wood’s C content fell from 70.85 to 54.16%, and 40.89%, after being inoculated with *A. flavus* and *P. chrysogenum*. The O atomic percentage rose from 28.78 to 45.19% and 52.43%, when inoculated with *A. flavus* and *P. chrysogenum*, respectively. Depending on how durable each wood was, The examined fungi were able to attack the two distinct types of wood in various deterioration patterns. *T. grandis* wood overtaken by the two molds under study appears to be a useful material for a variety of uses.

## Introduction

Numerous wood species, including *Ficus sycomorus*, *Cedrus libani*, *Quercus cerris*, *Zizyphus spina Christi*, and *Tamarix* sp. have been discovered in ancient Egyptian tombs^[1]^. The studied samples revealed varying preservation conditions in terms of carbohydrate and/or lignin loss despite their long-term burial in dry archaeological sites. High concentrations of soluble chemicals made it difficult to interpret the findings. These water-soluble substances contained depolymerized lignin or carbohydrates^[2]^. It is commonly known that wood, an organic natural material, is vulnerable to attack by fungi when the right conditions exist, such as when moisture content raises to 20% and temperature falls between 25 and 40 °C^[3–9]^.

When fungi invade wood, they consume its elemental composition and carbohydrates. Brown and white rot attack by depolymerizing the layers of the cell wall, whereas soft-rot fungi create cavities in the secondary wall^[3,10]^. Fungal growth and reproduction utilize starch and simple sugars contained in the structure, particularly in ray and axial parenchyma cells lumen, which results in structural changes of the wooden objects^[10]^. Fungi create extracellular enzymes, such as cellulase, xylanase, and α-l-arabinofuranosidase, as they multiply and colonize wood in a dynamic and competitive process^[11–16]^. They can also grow in wood’s longitudinal, radial, and tangential orientations^[17–19]^. The hyphae can pass between the wood rings, into the cell wall, between fibers, and through pits^[18,20–22]^.

The *Penicillium*, *Paecilomyces*, and *Aspergillus* genera of molds, can deteriorate wood and wood products^[7]^. Cellulase and other extracellular enzymes produced by *Penicillium* species^[23]^, break down pectin and xylan^[24]^. *Paecilomyces variotii*, the soft-rot fungus, produces amylase enzyme^[24]^. The xylanases enzyme^[24,25]^ and hydrolytic enzymes that can break down hemicelluloses and cellulose^[26]^, are produced by *Aspergillus* species. Although some molds, like *Trichoderma viride*, do not decompose wood, they do enzymatically consume the nutrients found in the parenchyma cells^[27]^.

After 4 years of colonization in *Acacia saligna* wood, *Aspergillus niger*, *A. flavus*, *Alternaria tenuissima*, *Fusarium culmorum*, and *T. harzianum* revealed checks and breaks inside the secondary cell wall regions, which were caused by the action of acids on polysaccharides^[28]^. *T. viride* and *A. alternata*, a surface mold fungi, have the ability to alter the ultrastructure of wood similarly to soft-rot fungi^[8]^.

Cellulase enzymes are secreted by the lignocellulolytic fungi of the genera *Aspergillus* and *Penicillium*, which hydrolyze cellulose to produce glucose, a monosaccharide molecule^[4]^. *Penicillium chrysogenum* is another fungus that rots wood; its hyphae are closely attached to the structure of the wood, and erosion troughs developed in the wood’s deteriorating cell walls in soil^[5,8]^. Amorphous cellulose and xylan (hemicellulose) breakdown in wood has been retained in dry conditions and over an extended length of time^[29]^. Dry ancient wood degrades more complexly than waterlogged wood^[2]^.

Among the traditional examination methods, Computed tomography (CT), one of the more common examination techniques, has the potential to be a tool for measuring the depth of fungal penetration in wood. CT generates cross-sectional (tomographic) images (virtual "slices") from various angles of specific areas, allowing a nondestructive closer look at fungal growth within wood structures^[30–32]^.

This research looked at how *Aspergillus flavus* and *Penicillium chrysogenum* affected *Ficus sycomorus* and *Tectona grandis* after being inoculated over a 36-month period.

## Materials and methods

### Wood samples

This study has complied with relevant institutional, national, and international guidelines and legislation. This study does not contain any studies with human participants or animals performed by any of the authors. Sapwood samples of *Ficus sycomorus*, a 25-year-old native tree species, and *Tectona grandis*, an imported wood^[33]^, were both used as sources of wood. In a wood workshop in Alexandria, Egypt, ten woodblock samples from *F. sycomorus* and *T. grandis* were prepared with dimensions of 0.5 × 1 × 2 cm, autoclaved at 121 °C and then dried in an oven at 103 ± 2 °C for 24 h^[34,35]^.

### Tested fungi

For the wood inoculation, two soft-rot fungi *Aspergillus flavus* and *Penicillium chrysogenum*, were used. These fungi were previously molecularly identified and deposited in the GenBank under the accession numbers LC325160, and LC325162, respectively.

### Inoculation of wood samples with the tested fungi

Each piece of wood sample was individually inoculated in Petri plates with media of potato dextrose agar (PDA) and fungus discs from *A. flavus* (ACC# LC325160), and *P. chrysogenum* (ACC# LC325162) measuring 5 mm in diameter^[9,36]^. Following this, fungi were cultured on wood blocks under experimental conditions (27 ± 2 °C and 70 ± 5% relative humidity (RH)), where the incubation lasted until the complete colonization of the fungus on the Petri dishes and wood block. After this time, Petri plates were covered a plastic wrap made of cling film and stored in a growth chamber for 36 months at the ambient conditions (27 ± 2 °C and 70 ± 5% RH)^[28]^. The inoculated woodblocks were then removed for additional examination.

### SEM–EDX examination of the inoculated wood samples

Using an environmental scanning electron microscope (ESEM) (Quanta FEG250; FEI Co., Hillsboro, OR, USA), the inoculated wood samples of *F. sycomorus* and *T. grandis* were analyzed for the fungal infestation (*A. flavus* and *P. chrysogenum*) on the surface and at 5-mm depth of the infected wood block. Energy dispersive spectroscopy (ESEM–EDS (Quanta FEG250; FEI Co., Hillsboro, OR, USA, with tungsten electron source, at 20 kV) was used to examine the variations in surface elemental compositions.

### CT scanning examination

The hyphal fungal infestation within the tested wood samples was examined by X-ray computed tomography (CT) scanning using a Toshiba Aquilion 16 CT Scanner, Tokyo, Japan. Using 3-D imaging, which is an image quality with surface shaded-renderings and volume-rendered 3-D images, datasets were displayed and images and videos were recorded. Distance measurements were made as the 3-D surface was zoomed in and out. The Aquilion 16 features 896 channels in 40 rows of solid-state detectors with a variety of slice thicknesses was used to test high image quality. In the x, y, and z directions, the system had a low-contrast resolution of 2 mm at 0.3% and a high-contrast resolution of 0.35 mm. The following values were used: voltage 120 kV, current 150 mA, timing 15.819 s, and thickness 0.5 × 16 mm^2^^[37]^. The CT number, which is expressed by brightness data in an image, is based on linear X-ray absorption coefficients^[38]^.

## Results and discussion

### SEM and CT scanning examination results of fungal inoculated *Ficus sycomorus* wood

**Figure 1:**
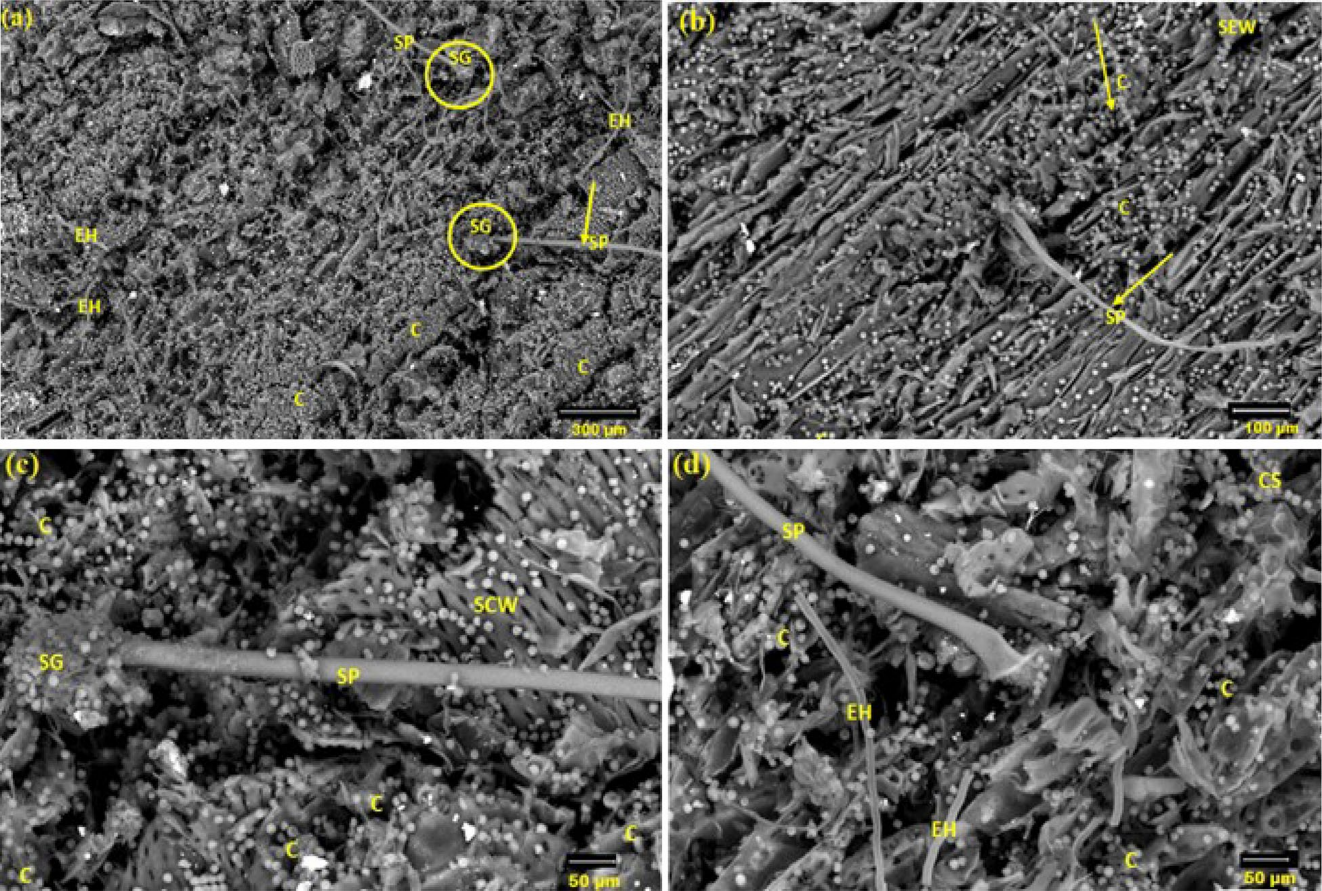
SEM images of wood samples from *F. sycomorus* that have been inhabited by the rapid growth of *A. flavus*. A number of aerial fungal mycelium are displayed in (**a**), conidia are distributed, sporangiophore and conidia are displayed, and the fungus’ exterior hyphae are displayed in (**b**–**d**). *SG* sporangium, *CS* conidiospores, *EH* external hyphae, *SP* sporangiophore, *SCW* secondary cell wall, *C* conidia.

On *Ficus sycomorus* wood, *Aspergillus flavus* growth was clearly visible in all samples (Fig. 1). A visual augmentation in conidia quantity was observed at different areas with Several aerial fungal mycelium and conidia covered the surface of *F. sycomorus* in various locations, causing a visible increase in conidia abundance (Fig. 1a and b). Additionally, conidiospores and mycelium quantity increased as a result of aberrant spore development along the hyphae in the basal mycelium (Fig. 1c and d).

**Figure 2:**
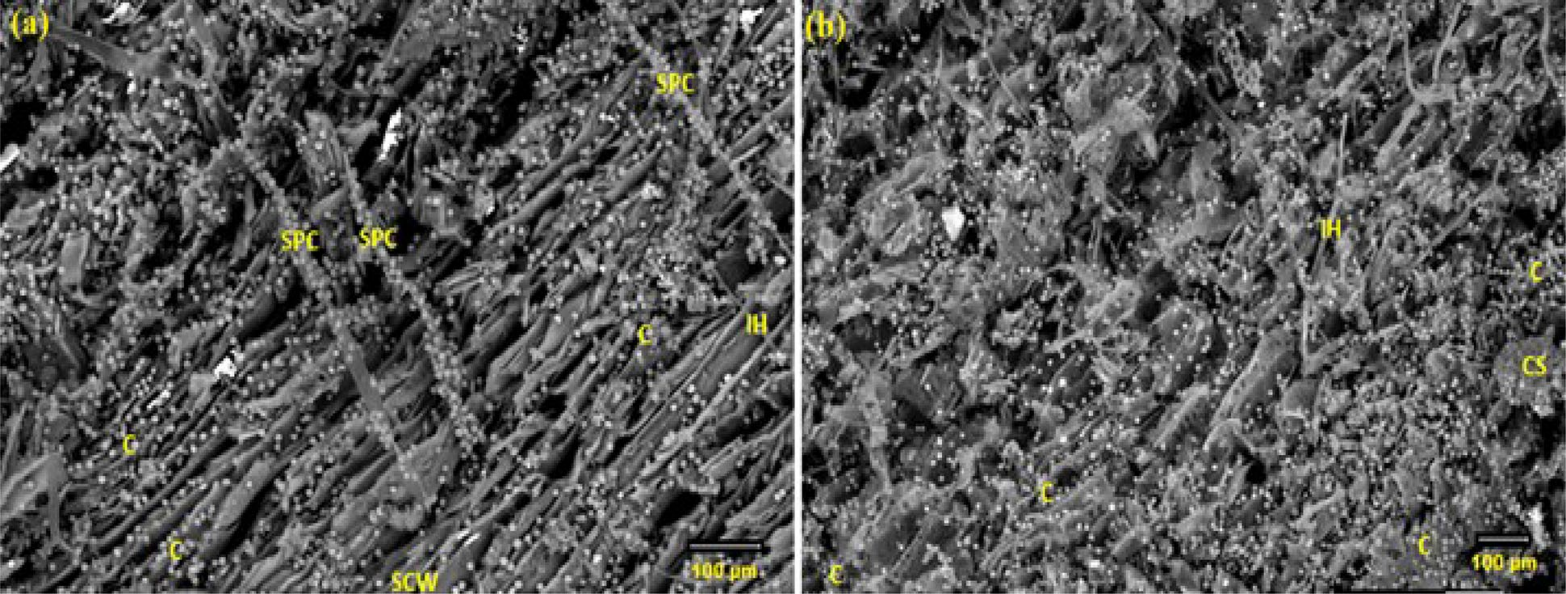
An SEM image of the infected *F. sycomorus* wood (5-mm depth) with A. flavus reveals the spore, the growth morphology of the hyphae within the wood fibers, and the penetration of hyphae inside the wood and within cells due to *A. flavus* infection. (**a**) shows the sticky conidia and (**b**) shows the distribution of conidia and internal hyphae. *IH* internal hyphae, *CS* conidiospores, *SPC* sporangiophores with sticky conidia, *SCW* secondary cell wall, *C* conidia.

**Figure 3:**
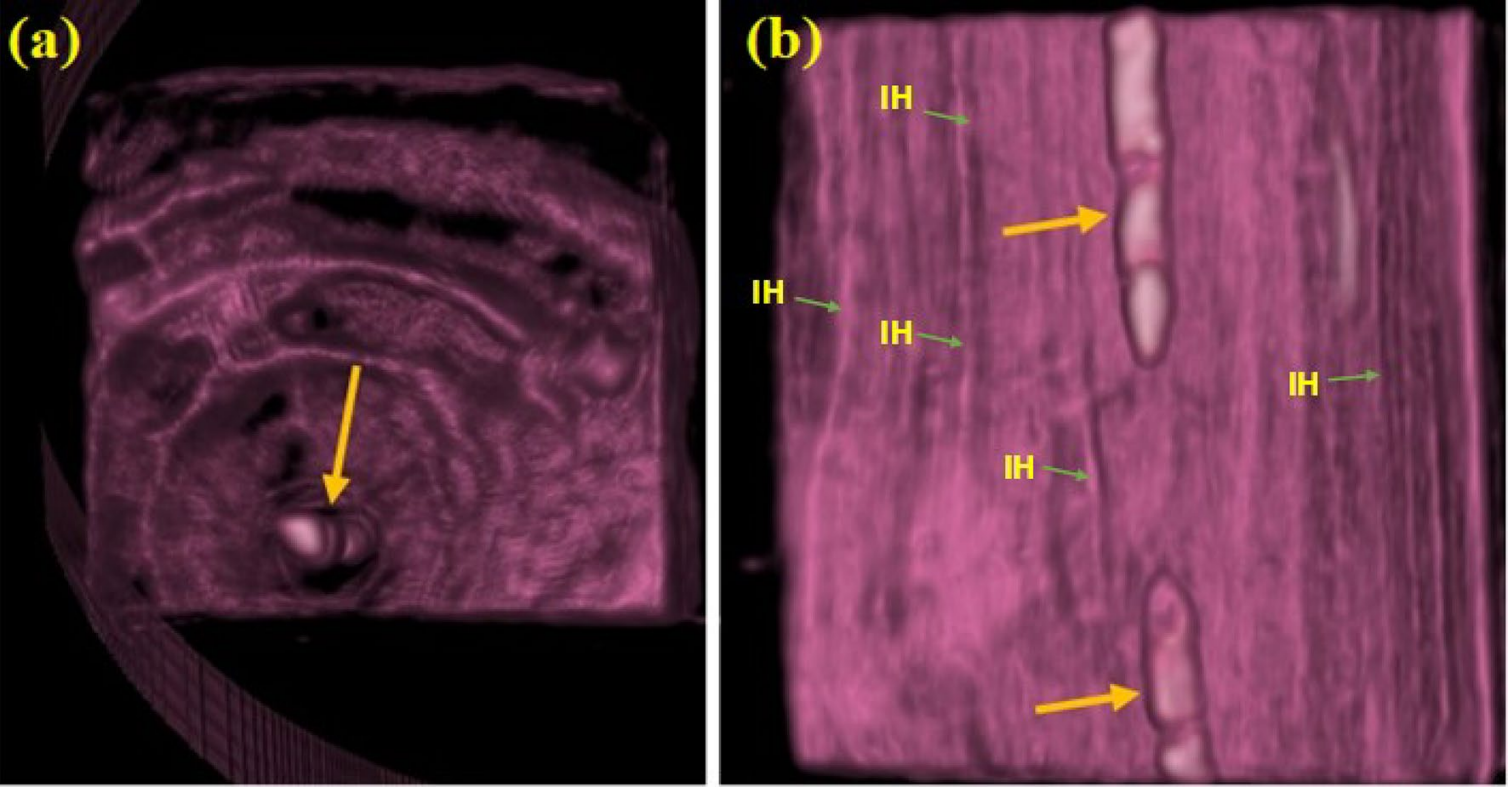
CT scan at cross-section (**a**) and longitudinal direction (**b**) of the inoculated *F. sycomorus* wood with *A. flavus* after 36 months; big arrows refer to insect-made pores; *IH* internal hyphae.

The SEM images of the analyzed *F. sycomorus* wood sample that was 5 mm deep and infected with *A. flavus* are displayed in Fig. 2a and b. The infected wood had *A. flavus* at its ideal density, which resulted in a notable increase in hyphal development and spore generation. After 36 months of incubation, *A. flavus* was detected inside the wood by CT scanning, as shown in Fig. 3.

The production of *A. flavus* conidial, however, may rise even below the surface, showing that *A. flavus* can rot *F. sycomorus* wood through the pits and within cell walls. Additionally, among the wood fibers, the spore and hyphae structure was visible. In a previous study, it was discovered that various molds, including *Botryodiplodia theobromae*, *Trichoderma longibrachiatum*, *A. candidus*, *A. ustus*, and *A. terreus* quickly degraded *F. sycomorus* wood^[39]^. In addition to the great porosity of the wood, which allowed these aerobic fungi to grow through it, the low level of antimicrobial chemicals in *F. sycomorus* may have contributed to the enormous development of *A. flavus*^[39]^.

**Figure 4:**
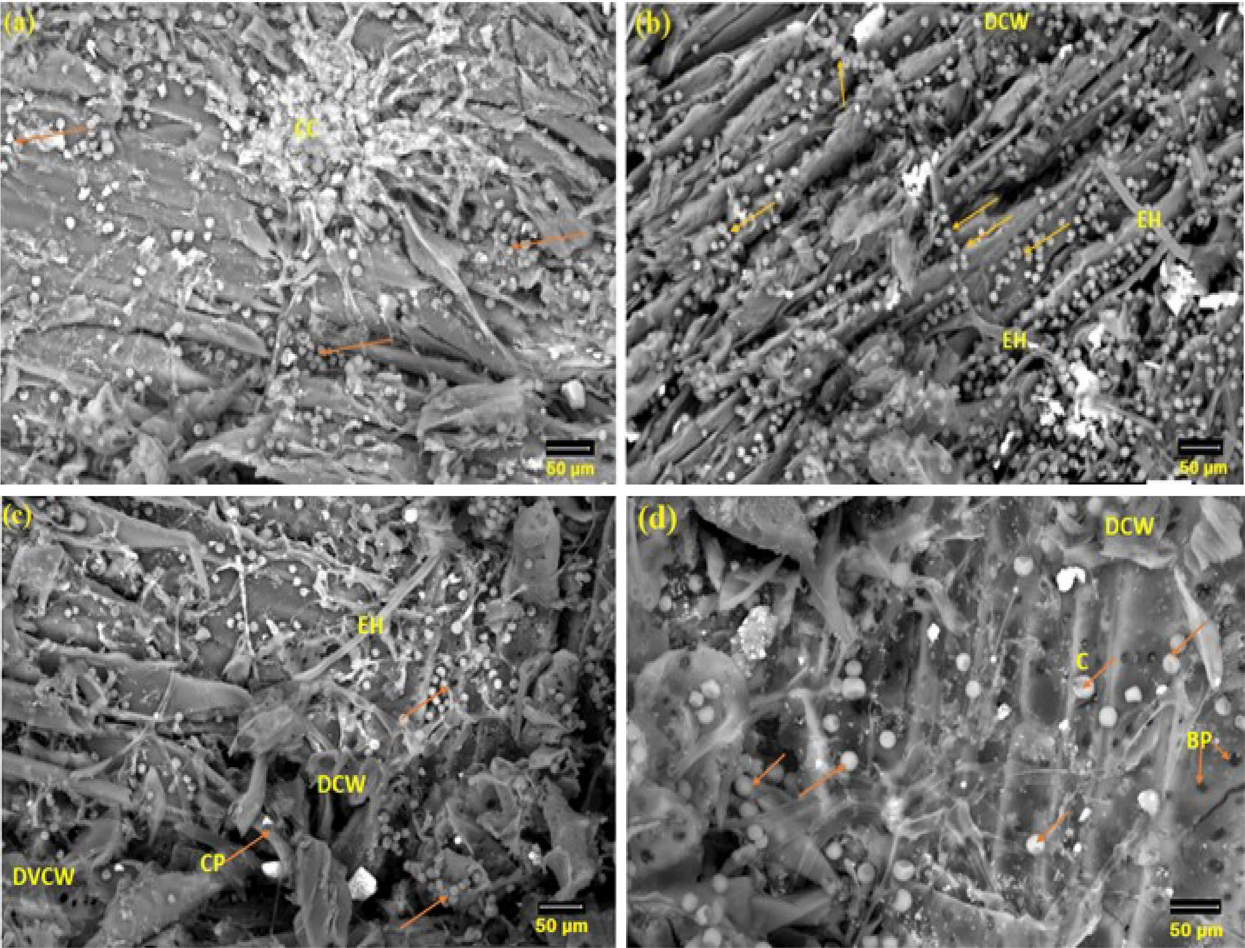
SEM surface features of *F. sycomorus* wood as inoculated with *P. chrysogenum* for 36 months, (**a**) shows the cluster of conidia, (**b**) shows the external hyphae with conidia, (**c**) shows the degraded vessel cell wall, and (**d**) shows the degraded cell wall. Arrows and circles refer to the intensive growth of *P. chrysogenum* (*CC* cluster of conidia, *DCW* degraded cell wall, *EH* external hyphae, *C* conidia, *CP* conidiophores, *DVCW* degraded vessel cell wall).

Figure 4 depicts the SEM morphology of *P. chrysogenum* colonies on wood after 36 months at 27 ± 2 °C. It was possible to see *P. chrysogenum*, which had peridial hyphae with thick-walled dichotomously branching walls and a short, bifurcating appendage resembling a spine. The center displays a gymnothecium of the globose open reticulum type with ascospore mass. Ascospores and developing asci are seen. It is common to find tiny, radially projecting peridial appendages that resemble deer antlers.

In the wood of *F. sycomorus*, *P. chrysogenum* produced hair baits (Fig. 4a). The ascomata appeared to have septate, branching, and thick-walled peridial hyphae, but lacked the distinctive boathook-shaped appendages. Globose- to subglobose asci were present. Under SEM, the spores were seen to be irregular and rough (Fig. 4b through d).

In addition to severely degraded and weakened cell walls, SEM images of *F. sycomorus* wood degradation after 36 months also revealed colonized hyphae in cell walls. The total disintegration of the cell wall and vessels may be linked to the severe chemical materials degradation of the cell wall^[40–43]^. As previously indicated, After 3 months of incubation with *A. niger*, identifiable notches of cell wall erosion and cavities created by fungal hyphae within the cell walls were discovered in wood., while *P. chrysogenum* only created erosion troughs formed in cell walls while colonizing *F. sycomorus* wood^[5]^.

**Figure 5:**
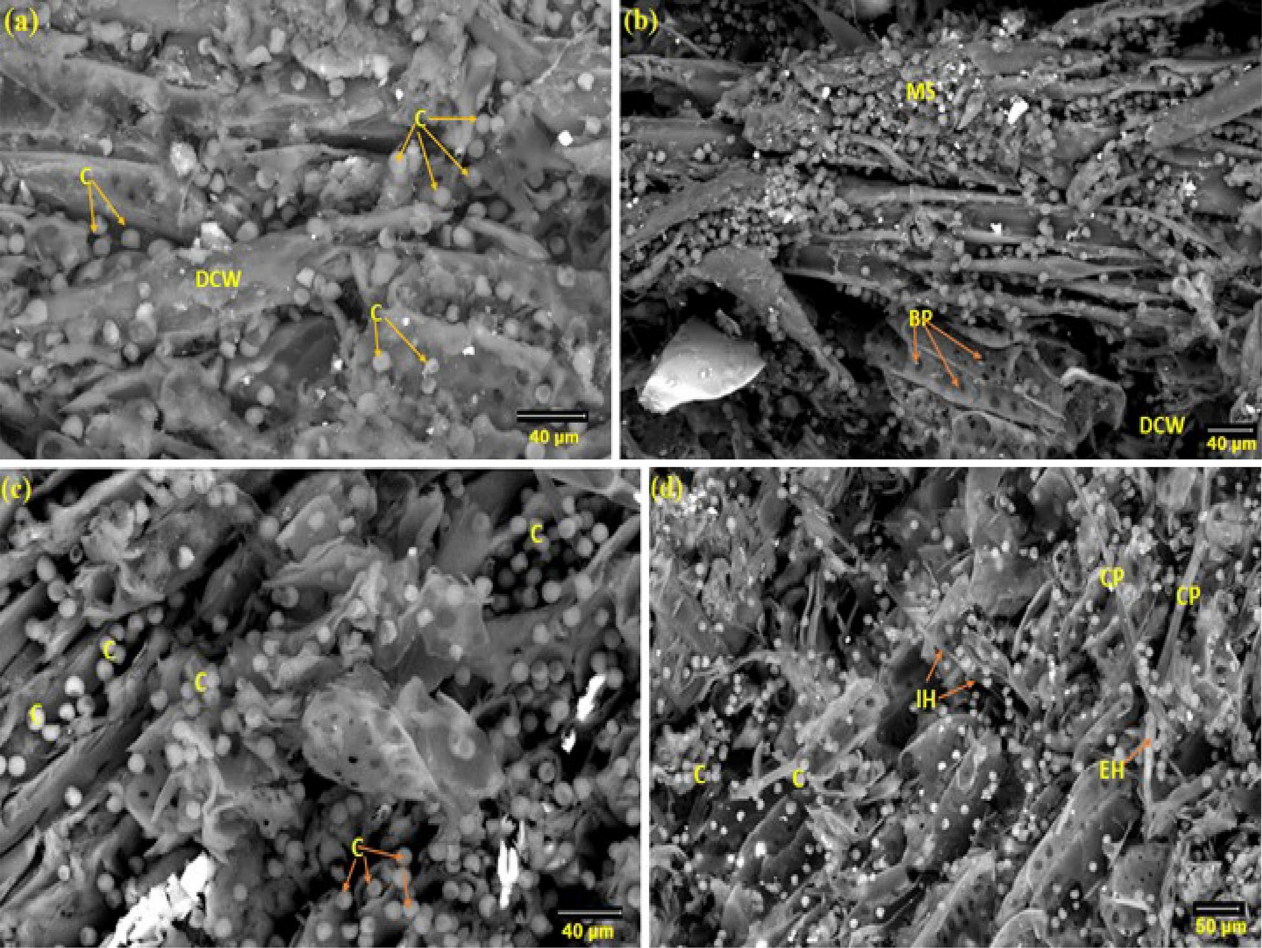
SEM micrograph of *F. sycomorus* after cutting 5-mm from infected wood and showing enlargement and deterioration of pits and detachment within cells due to *P. chrysogenum* attack and the spores and hyphae growth morphology are observed within the wood fibers: (**a**) shows the distribution of conidia and the degraded cell wall, (**b**) shows of mass of conidia, (**c**) shows the distribution of conidia, and (**d**) shows the Internal hyphae of *P. chrysogenum*. *MS* mass of conidia, *DCW* degraded cell wall, *EH* external hyphae, *IH* internal hyphae, *C* conidia, *CP* conidiophores.

**Figure 6:**
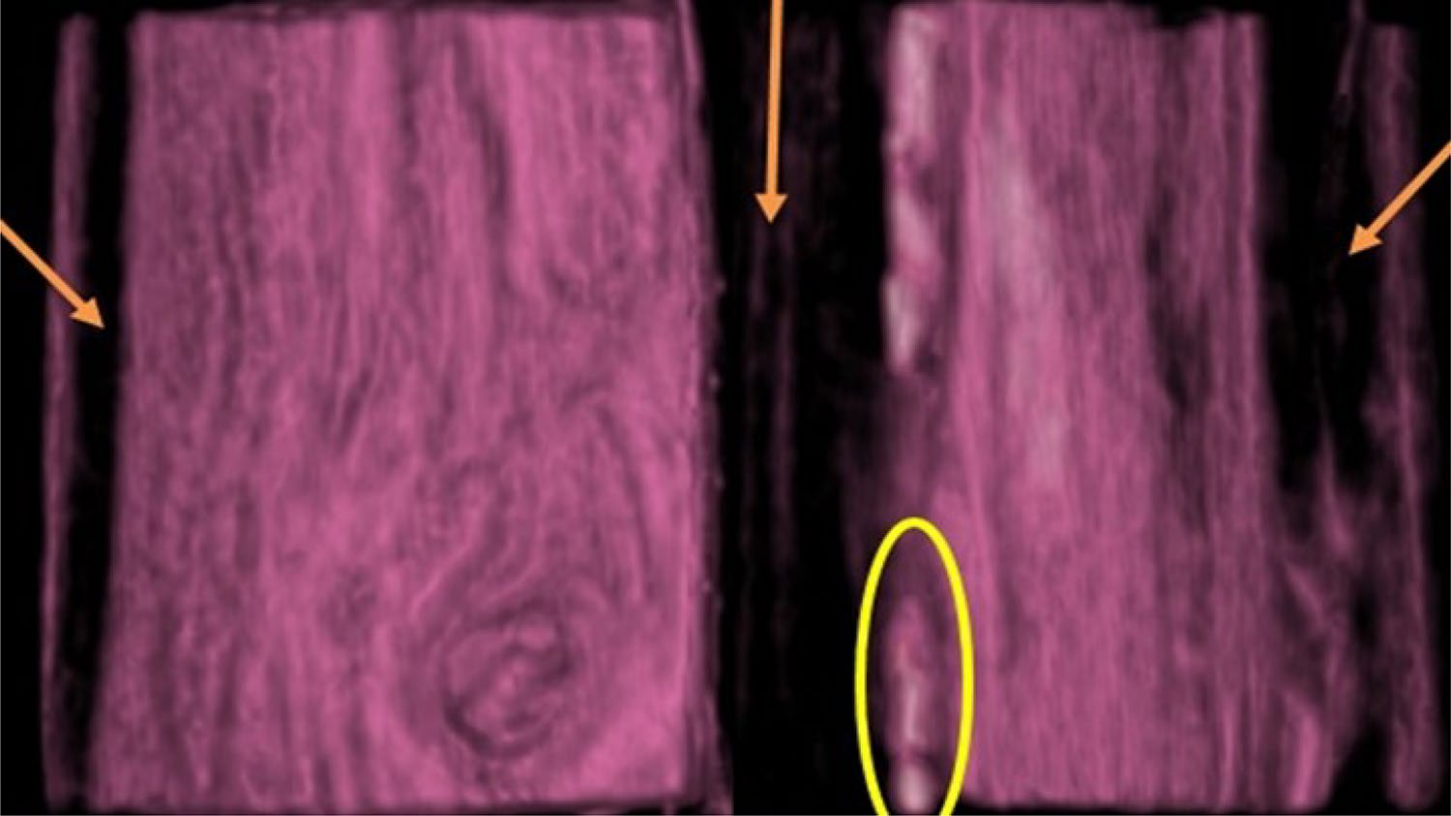
CT-scanningof *F. sycomorus* wood inoculated with *P. chrysogenum* for 36 months; arrows refer to the deterioration found in the wood after this long-term infection. The three arrows and circle depict the shapes of the longitudinal holes.

SEM images of a 5-mm-deep incision in the infected wood were taken to demonstrate the extent of *P. chrysogenum* inside *F. sycomorus* wood (Fig. 5a through d). Images revealed *P. chrysogenum* inside wood had grown enormously. After 36 months of incubation, a CT scanning (Fig. 6) of the wood demonstrated *A. flavus* penetration growth.

The SEM images of wood samples from *F. sycomorus* showed that the cell walls were distorted, had some structures holes, and were missing entire wood cells. These are the results of the development of fungus hyphae and spores^[29]^. Cell wall layers have likely detached and separated as a result of soft rot fungal growth^[20,44]^. Despite poor structural preservation, a fungal microbial degradation was discovered in archeological *F. sycomorus* wood^[2]^. After 4 months of incubation with *Penicillium chrysogenum*, the secondary wall layers of *F. sycomorus* wood were disrupted as a result of severe cell wall breakdown^[5]^.

### SEM and CT scanning examination of incubated *Tectona grandis* wood

**Figure 7:**
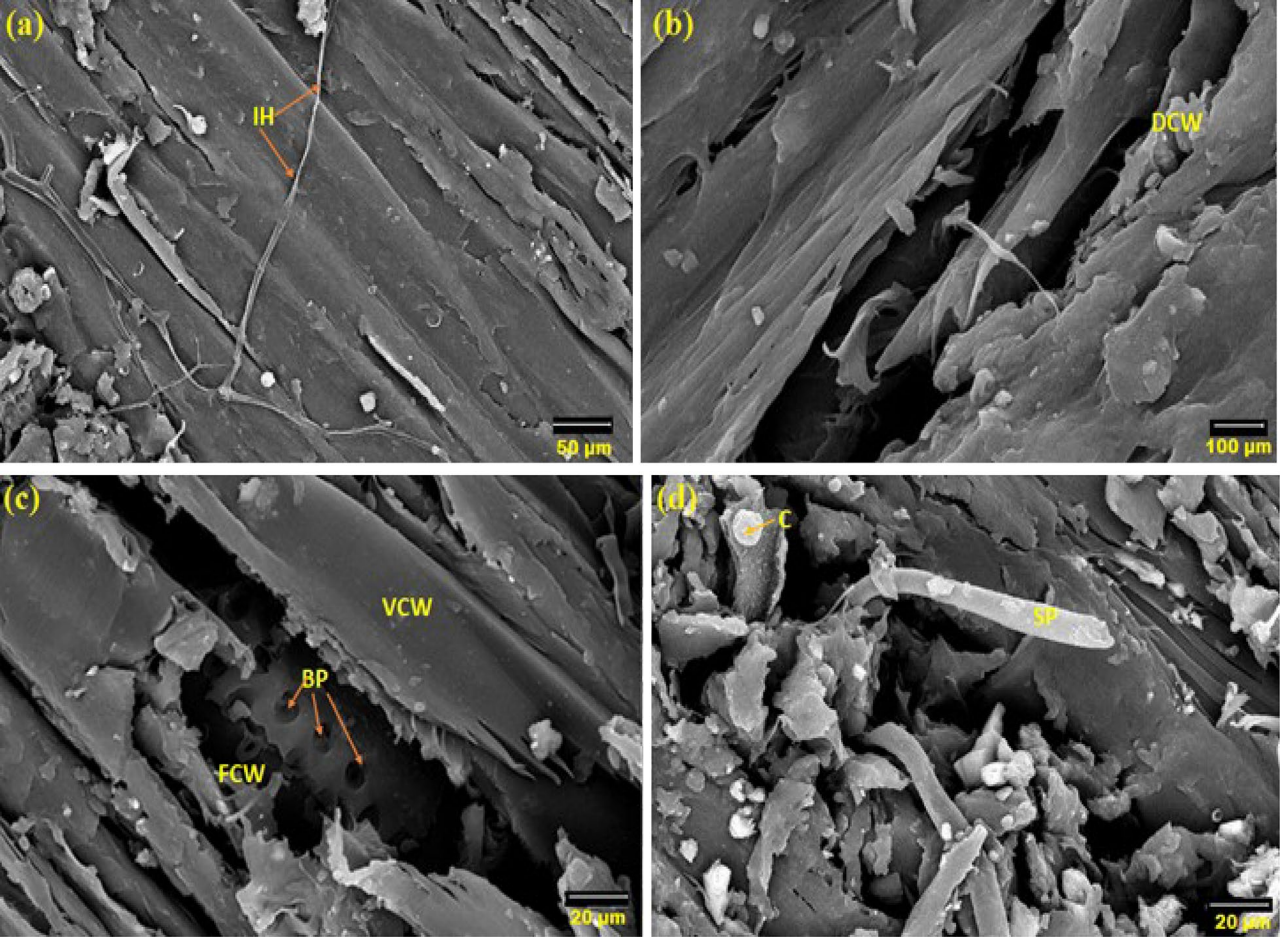
SEM images of teak wood that was surface-exposed to A. flavus for 36 months (**a**,**b**) and after cutting of 5-mm from infected wood (**c**,**d**). *IH* internal hyphae, *SP* sporangiophore, *C* conidium: *FCW* fiber cell wall, *VCW* vessel cell wall, *BP* bordered pits.

**Figure 8:**
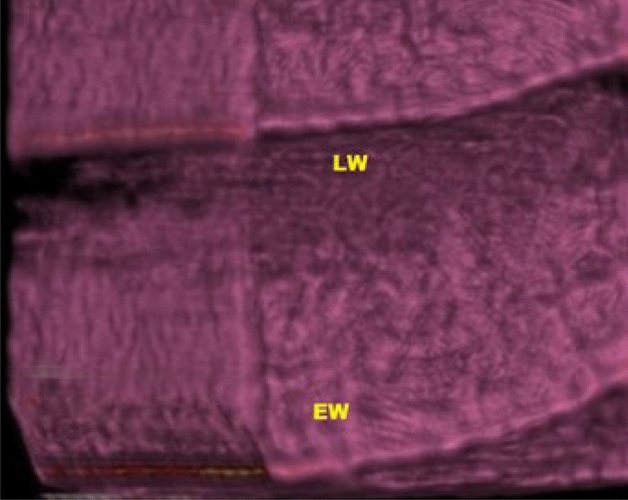
CT imaging of teak wood exposed to *A. flavus* after 36 months of incubation; *LW* latewood, *EW* early wood.

In all teak wood samples, the growth of *A. flavus* was minimized (Fig. 7a and b). The spore loss is visible in the SEM images. The branching and decay are the two main modifications to the hyphal morphology. According to Fig. 7c and d, phenolic and other aromatic antimicrobial chemicals prevent the growth of *A. flavus* on teak wood. Even after 36 months of incubation, the CT scanning (Fig. 8) supported the resistance patterns of teak wood to the spread of *A. flavus*.

**Figure 9:**
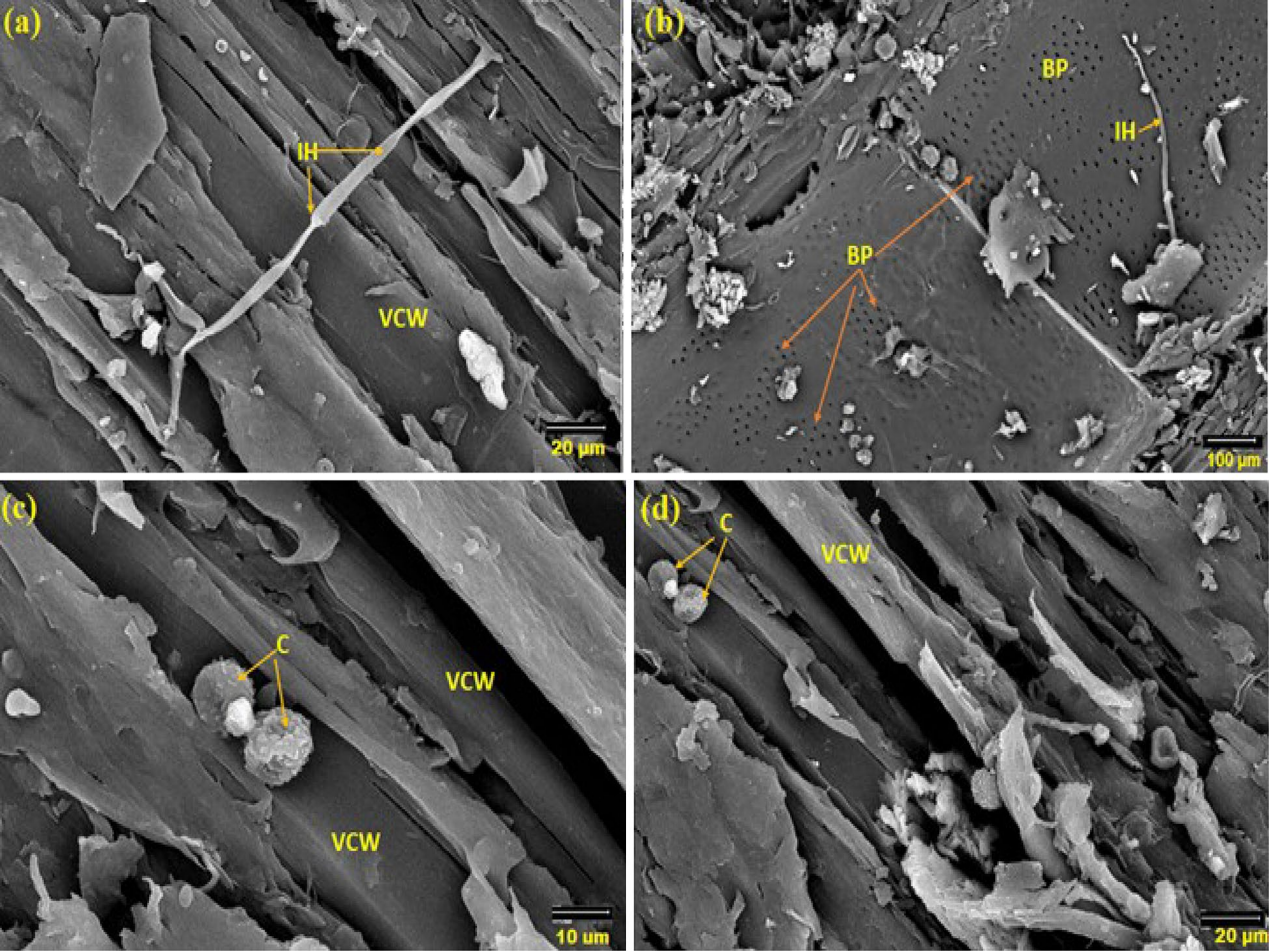
SEM micrograph of teak wood exposed to *P. chrysogenum* for 36 months on the surface (**a**,**b**) and at 0.5-mm depth of wood (**c**,**d**). *IH* internal hyphae, *C* conidium, *VCW* vessel cell wall, *BP* bordered pit.

After 36 months on incubation, teak wood infected with *Penicillium chrysogenum* showed reduced mycelial growth (Fig. 9a through d). These findings suggested that the conidial production of *P. chrysogenum* might not grow in teak wood due the presence of phenolic and other aromatic antimicrobial compounds, such as anthraquinines and tectoquinones^[45–50]^. The chemical compounds in the wood changed the morphology of *P. chrysogenum*, colony morphology, and multicellular clumps that lost the spore.

**Figure 10:**
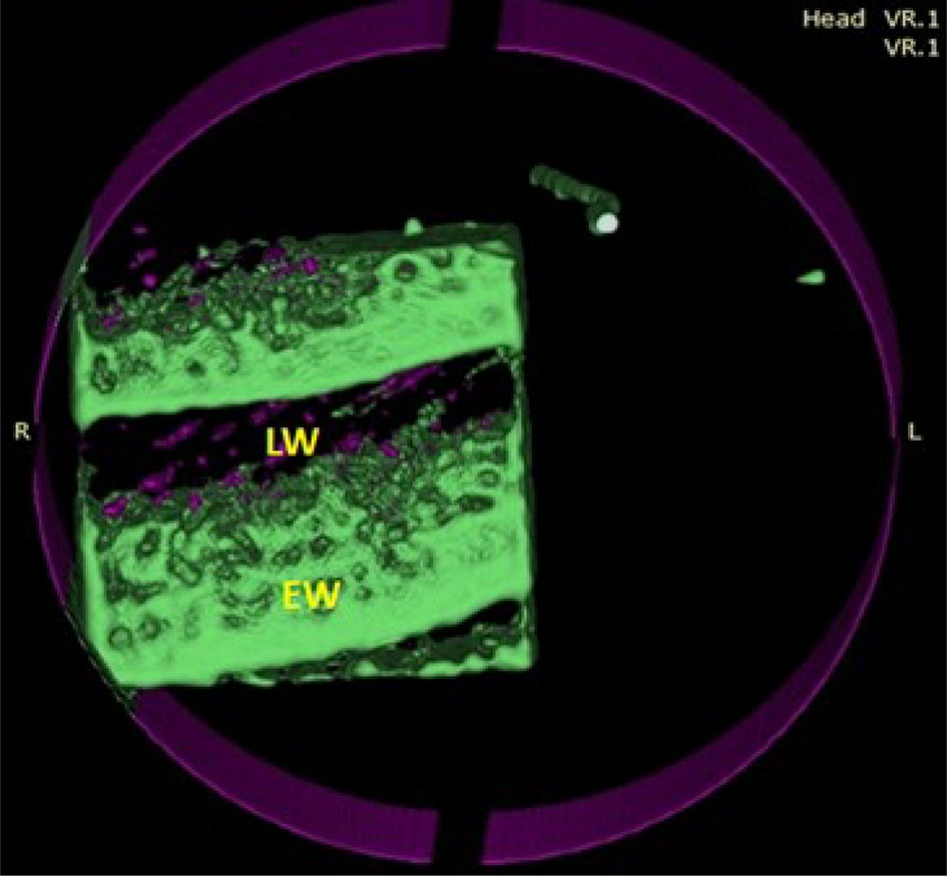
CT scanning of teak wood infected by *P. chrysogenum* after 36 months of the artificial inoculation. *LW* latewood, *EW* early wood.

It was discovered that the size of the mycelial pellets had significantly decreased, the cell wall had absorbed, and the shape of the mycelial pellets had changed. Nearly no growth indicators of *P. chrysogenum* were visible on the surface and core of the wood during the CT scanning (Fig. 10).

## EDX measurements

### Elemental composition changes of *Ficus sycomorus* wood

**Figure 11:**
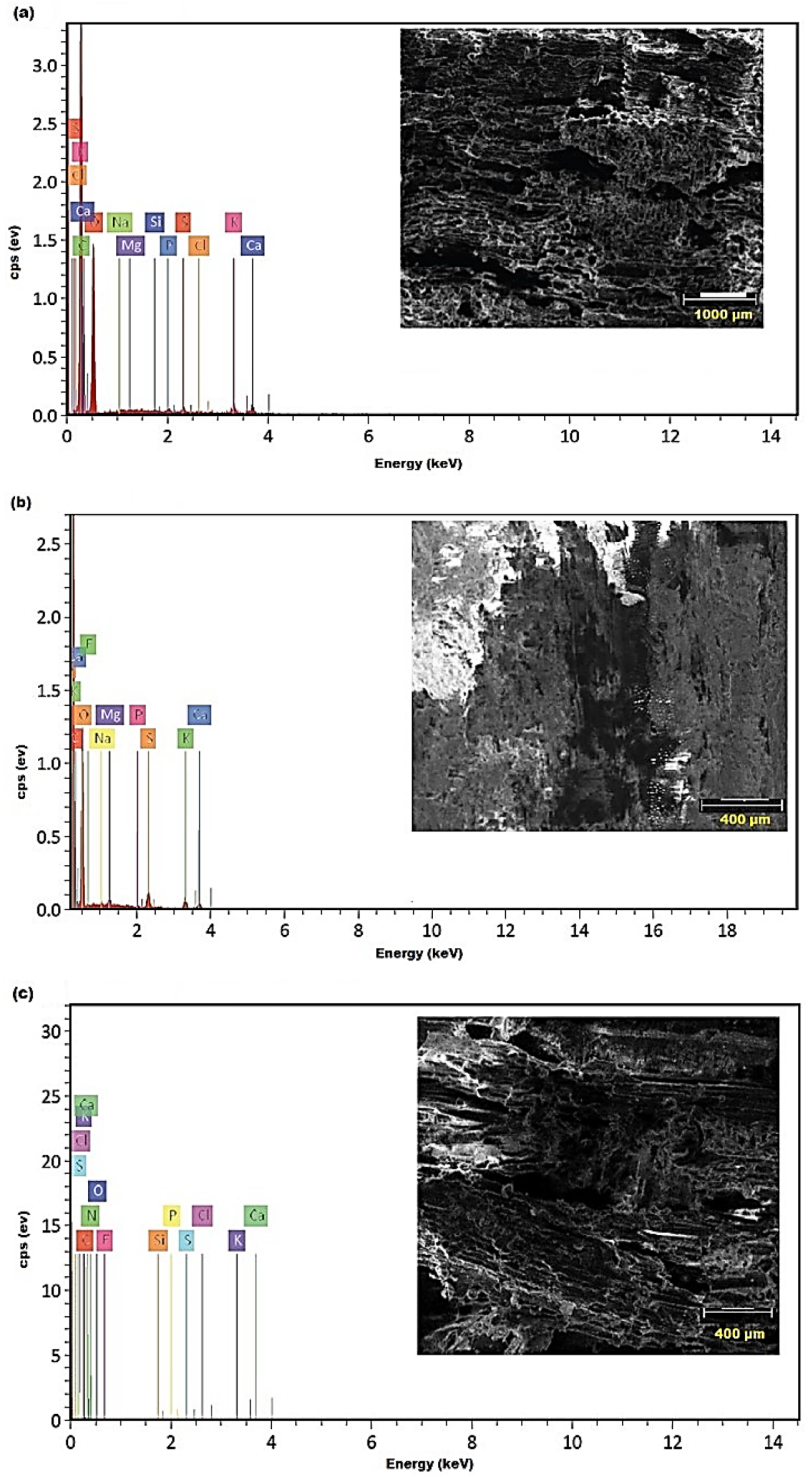
EDX spectral analysis of the elemental composition of un-inoculated *F. sycomorus* wood (**a**) and inoculated with *A. flavus* (**b**) and *P. chrysogenum* (**c**).

Incubated *F. sycomorus* wood with *A. flavus* and *P. chrysogenum* for 36 months is shown in Fig. 11 to have different elemental compositions from un-inoculated wood. In the un-inoculated wood (Fig. 11a and Table 1), The atomic percentages of C and O in the un-inoculated wood were 61.69%, and 37.81%, respectively, while they changed to 59.33% for C and 39.59% for O in the inoculated wood samples with *A. flavus* (Fig. 11b and Table 2). The C and O atomic percentages in wood samples inoculated with *P. chrysogenum* for 36 months dropped to 58.43%, and 26.34%, respectively (Fig. 11c and Table 3).

**Table 1:** EDX elemental compositions of un-inoculated *Ficus sycomorus* wood.

Element	At. no	Netto	Mass (%)	Mass norm. (%)	Atom (%)
Carbon	6	15,310	54.33	54.33	61.69
Oxygen	8	7183	44.36	44.36	37.81
Calcium	20	583	0.53	0.53	0.18
Potassium	19	421	0.29	0.29	0.10
Sulfur	16	240	0.20	0.20	0.08
Phosphorus	15	170	0.13	0.13	0.06
Silicon	14	83	0.05	0.05	0.02
Chlorine	17	56	0.04	0.04	0.02
Magnesium	12	43	0.04	0.04	0.02
Sodium	11	29	0.04	0.04	0.02

**Table 2:** EDX elemental compositions of *Ficus sycomorus* wood inoculated with *Aspergillus flavus*.

Element	At. no	Netto	Mass (%)	Mass norm. (%)	Atom (%)
Carbon	6	11,702	51.46	51.46	59.33
Oxygen	8	5765	45.75	45.75	39.59
Calcium	20	342	1.02	1.02	0.35
Potassium	19	504	0.95	0.95	0.34
Sulfur	16	763	0.54	0.54	0.23
Magnesium	12	228	0.12	0.12	0.07
Sodium	11	120	0.08	0.08	0.05
Phosphorus	15	88	0.06	0.06	0.02
Fluorine	9	2	0.02	0.02	0.01

**Table 3:** EDX elemental compositions of *Ficus sycomorus* wood inoculated with *Penicillium chrysogenum*.

Element	At. no	Netto	Mass (%)	Mass norm. (%)	Atom (%)
Carbon	6	651	5.88	47.97	58.43
Oxygen	8	134	3.53	28.81	26.34
Nitrogen	7	15	1.04	8.52	8.90
Potassium	19	252	0.69	5.60	2.09
Calcium	20	221	0.61	4.96	1.81
Fluorine	9	10	0.21	1.72	1.32
Sulfur	16	24	0.11	0.90	0.41
Chlorine	17	20	0.09	0.73	0.30
Phosphorus	15	12	0.06	0.50	0.23
Silicon	14	8	0.04	0.31	0.16

The un-inoculated wood contained 0.10% atomic percentage of K. In the infected wood with *A. flavus*, and *P. chrysogenum*, this climbed to 0.34% and 2.09%, respectively. Additionally, the Ca content rose from 0.18% in the control wood sample to 0.35% with *A. flavus* infected wood sample and to 1.81% in wood sample inoculated with *P. chrysogenum*.

### Elemental composition changes of teak wood

**Figure 12:**
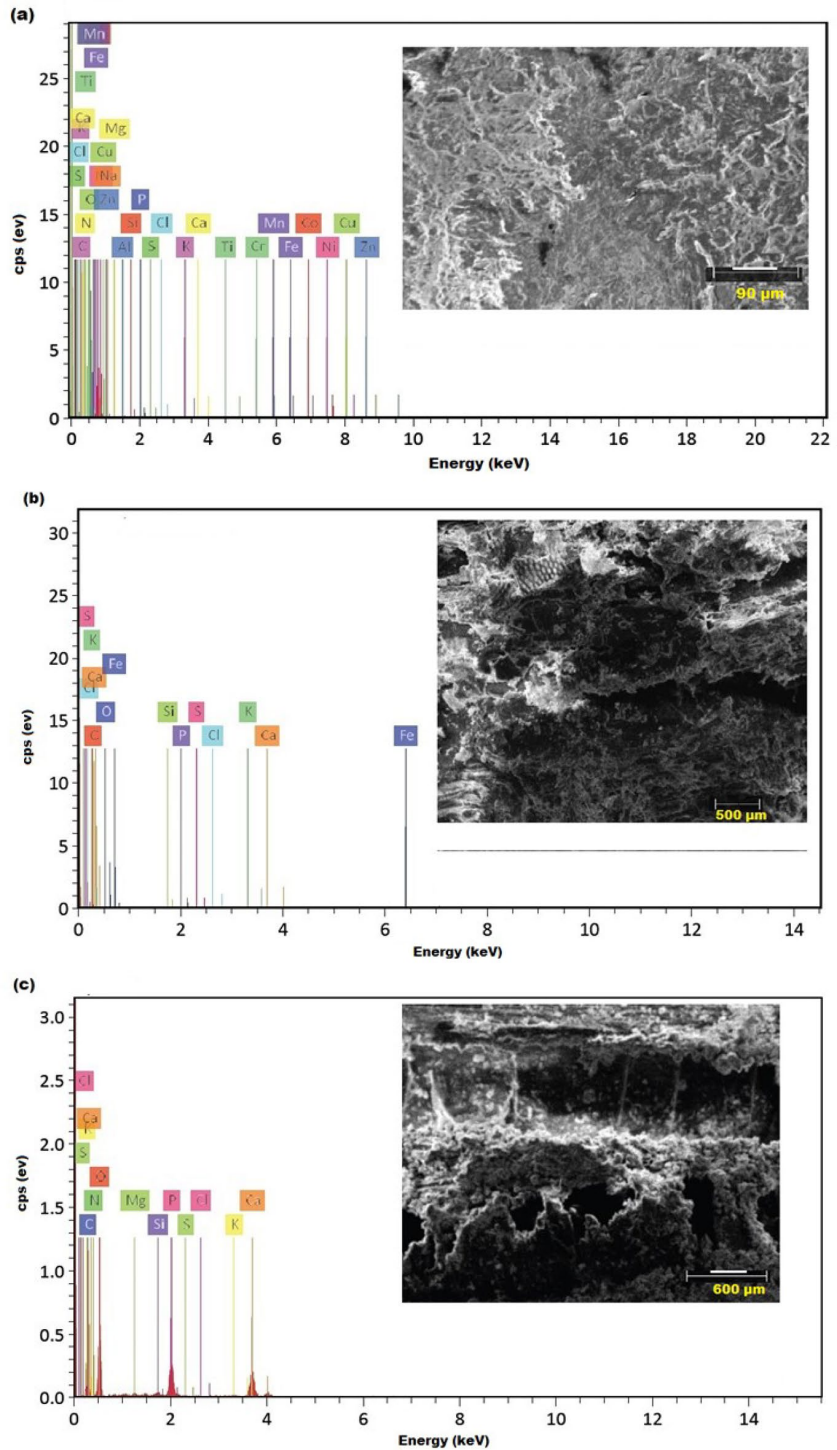
The elemental composition of un-inoculated teak wood analyzed using EDX spectroscopy. (**a**), inoculated with *A. flavus* (**b**) and inoculated with *P. chrysogenum* (**c**).

As compared to the un-inoculated sample, Fig. 12 illustrates the changes in the elemental composition of teak wood that was incubated for 36 months with *A. flavus* and *P. chrysogenum*. In the un-inoculated wood sample, C and O are the two most prevalent elements with atomic percentages of 70.85% and 28.78%, respectively (Fig. 12a and Table 4). As teak wood was inoculated with *A. flavus* (Fig. 12b and Table 5) and *P. chrysogenum* (Fig. 12c and Table 6) for 36 months, the atomic percentage of the C element decreased to 54.16% and 40.89%, respectively, while the O atomic percentage increased to 45.19% and 52.43%, respectively.

**Table 4:** EDX elemental compositions of un-inoculated teak wood.

Element	At. no	Netto	Mass (%)	Mass norm. (%)	Atom (%)
Carbon	6	12,351	64.21	64.21	70.85
Oxygen	8	3044	34.74	34.74	28.78
Calcium	20	267	0.41	0.41	0.14
Iron	26	46	0.24	0.24	0.06
Potassium	19	67	0.07	0.07	0.02
Sodium	11	43	0.05	0.05	0.03
Fluorine	9	5	0.05	0.05	0.03
Sulfur	16	52	0.04	0.04	0.02
Magnesium	12	43	0.04	0.04	0.02
Cobalt	27	4	0.03	0.03	0.01
Silicon	14	47	0.03	0.03	0.01
Chlorine	17	30	0.02	0.02	0.01
Manganese	25	5	0.02	0.02	0.01
Nickel	28	2	0.02	0.02	0.00
Aluminium	13	21	0.02	0.02	0.01
Phosphorus	15	17	0.01	0.01	0.00
Nitrogen	7	0	0.00	0.00	0.00
Titanium	22	0	0.00	0.00	0.00

**Table 5:** EDX elemental compositions of teak wood inoculated with *Aspergillus flavus*.

Element	At. no	Netto	Mass (%)	Mass norm. (%)	Atom (%)
Oxygen	8	7196	51.57	51.73	45.19
Carbon	6	10,318	46.40	46.54	54.16
Calcium	20	413	0.82	0.82	0.29
Silicon	14	415	0.33	0.33	0.16
Potassium	19	166	0.28	0.28	0.10
Chlorine	17	104	0.12	0.13	0.05
Iron	26	19	0.10	0.10	0.02
Sulfur	16	63	0.06	0.06	0.03
Phosphorus	15	6	0.01	0.01	0.00

**Table 6:** EDX elemental compositions of teak wood inoculated with *Penicillium chrysogenum*.

Element	At. no	Netto	Mass (%)	Mass norm. (%)	Atom (%)
Oxygen	8	3179	47.98	53.83	52.43
Carbon	6	1936	28.09	31.51	40.89
Calcium	20	1978	7.11	7.97	3.10
Phosphorus	15	2081	4.96	5.56	2.80
Magnesium	12	106	0.33	0.37	0.24
Nitrogen	7	6	0.25	0.28	0.31
Silicon	14	114	0.22	0.25	0.14
Potassium	19	41	0.12	0.13	0.05
Sulfur	16	19	0.05	0.06	0.03
Chlorine	17	10	0.03	0.04	0.02

The authors found that there were both declines and increases in the concentration of various key components from the analytical results of the elemental compositions of the inoculated wood with the two molds under study. Molds are typically discovered and grow in moisture-damaged wood^[51]^, create colored spores and large amounts of pigment on the surfaces of wood, which decrease the quality of the wood^[52,53]^, but do not influence the strength of the wood^[54]^.

The carbon-rich components of wood are reported to be metabolized by molds and fungi that disintegrate wood. This produces massive fruiting structures of fungi, which release a huge amount of spores into the natural environment^[10,55]^. Molds that are unable to depolymerize the primary chemical polymers of wood (cellulose, lignin and hemicelluloses) can consume the sugars and starches found in ray and axial parenchyma cells lumen^[56]^. Through pores and pits, The hyphae of the fungus can enter the cell walls through holes and crevices^[57]^.

The two mold fungi-inoculated woods showed that the C element content was lower than it was in the control sample. This outcome is consistent with previous work^[10]^. Additionally, it was found that the molds absorbed the C sources^[58]^. When growing on *Fagus sylvatica* wood, molds like *P. selerotigenum* and *A. niger* consume a lot of C, but *P. selerotigenum* consumes a lot of C content when growing on *Juglans nigra* wood. In contrast, minimal change in C content of *P. rigida* wood was observed when colonized by *P. selerotigenum*, *Paecilomyces variotii*, and *A. niger*, on the other hand, caused no change in the C content of *P. rigida* wood^[7]^.

According to the tests of adhesion in the study of Soumya et al.^[59]^, *P. chrysogenum* was unable to adhere to the cedar wood substrate, although *P. granulatum*, *P. crustosum*, and *P. commune* were able to do so, contrary to what was theoretically expected. The development of *P. chrysogenum* PCL501 on wood waste results in the production of the xylanase enzyme, which is most strongly induced by the carbon source^[60]^. When cultured on a bran-wood flour-olive oil or a bran-soy bean media, the water-soluble enzyme (Lipase) generated by *P. oxalicum* and *A. flavus* was capable of hydrolyzing the olive oil^[61]^.

The lignin structure in agricultural lignocellulosic wastes was discovered to be degraded by the strain of *A. flavus* EGYPTA5, which secretes lignin peroxidases, nitrate reductase, laccase, polyphenol oxidase, and cellulase enzymes, without changing the concentration of cellulose^[62]^. It was discovered that several *A. flavus* fungal isolates produced cellulase-free xylanase in a variety of soil environments, including manures, dead and decaying wood, and soil samples^[63]^. *A. flavus* produced the most cellulase enzyme when it was cultivated on wood sawdust, according to study^[64]^. *A. flavus* was isolated from its natural environment including wastewater, rotting wood, wheat straw, and field soil samples, and it produced laccase enzyme^[65]^.

Both SEM and CT scanning examinations confirmed the growth of molds on the studied wood samples on the surface and core showing the structural growth of fungi.

## Conclusions

The dispersion of the two molds’ fruiting structures, spores, and hyphae that covered the damaged wood surfaces after 36 months of incubation could be clearly seen by the examination instruments SEM–EDX and CT scanning. According to the study, the carbon-rich components of the examined *Ficus sycomorus* and *Tectona grandis* woods are metabolized proportionately by *Aspergillus flavus* and *Penicillium chrysogenum*. The findings supported the long-term durability and the non-durability phenomena of *Tectona grandis* and *Ficus sycomorus* woods, respectively. Finally, the surface and core of the analyzed wood samples showed structural growth of fungi, which was validated by SEM and CT scanning studies.

## Data Availability

All data generated or analyzed during this study are included in this published article.
